# LncRNA Structural Characteristics in Epigenetic Regulation

**DOI:** 10.3390/ijms18122659

**Published:** 2017-12-08

**Authors:** Chenguang Wang, Lianzong Wang, Yu Ding, Xiaoyan Lu, Guosi Zhang, Jiaxin Yang, Hewei Zheng, Hong Wang, Yongshuai Jiang, Liangde Xu

**Affiliations:** 1 College of Bioinformatics Science and Technology, Harbin Medical University, Harbin 150081, China; wangcg@hrbmu.edu.cn (C.W.); wanglz@hrbmu.edu.cn (L.W.); dingyu@hrbmu.edu.cn (Y.D.); luxy@hrbmu.edu.cn (X.L.); zhanggs@hrbmu.edu.cn (G.Z.); yangjx@hrbmu.edu.cn (J.Y.); zhenghw@hrbmu.edu.cn (H.Z.); 2 Training Center for Students Innovation and Entrepreneurship Education, Harbin Medical University, Harbin 150081, China

**Keywords:** lncRNA structure, epigenetic regulation, RNA-protein binding motif, regulation protein recruit

## Abstract

The rapid development of new generation sequencing technology has deepened the understanding of genomes and functional products. RNA-sequencing studies in mammals show that approximately 85% of the DNA sequences have RNA products, for which the length greater than 200 nucleotides (nt) is called long non-coding RNAs (lncRNA). LncRNAs now have been shown to play important epigenetic regulatory roles in key molecular processes, such as gene expression, genetic imprinting, histone modification, chromatin dynamics, and other activities by forming specific structures and interacting with all kinds of molecules. This paper mainly discusses the correlation between the structure and function of lncRNAs with the recent progress in epigenetic regulation, which is important to the understanding of the mechanism of lncRNAs in physiological and pathological processes.

## 1. Introduction

Recently, the rapid development and application of high-throughput sequencing technology further steadies the key situation of the connecting link of RNA in the focal dogma [1,2]. Through genome-wide human transcriptional studies, more and more new non-coding RNAs have been found [3]. Emerging evidence shows that the finding of long non-coding RNAs (lncRNAs), a less characterized class of molecules that are greater than 200 nucleotides (nt) in length, lead to the gene number duplicated in the databases [4]. The noncode database includes 73,370 lncRNAs from 1229 organisms, up to now [5], More than 50,000 new RNA transcripts have been found for the human genome in different tissue and cell types, most of which are lncRNAs, as well as plant and animal studies [6,7]. All of those lncRNAs enrich the biological diversity of the ecosphere, and complicate the studies of cell regulated mechanisms.

LncRNAs often form relatively stable secondary and higher structures, making them have the ability to participate in cellular organization and regulation, such as DNA replication, RNA transcription, protein translation, cell development, and cell differentiation [8,9]. The complex structural features also give the potential for lncRNA in epigenetic process. LncRNAs could influence changes in the gene expression or chromosome activity by a series of mechanisms, such as inducing DNA and protein modification, recruiting protein, and RNA interaction etcetera [9,10]. Some representative lncRNAs and their epigenetic functions are listed in Table 1. Combining the epigenetic function with the plasticity, variability, and tissue specificity, lncRNAs are regarded to be vital factors and biomarkers in disease genesis and diagnosis. Studies have demonstrated that the dysregulation of lncRNAs can lead to numerous types of diseases, including cancer, diabetes, cardiovascular disease, and some other complex disorders [11,12,13].

This review will mainly consider the correlations of lncRNA structure and function in epigenetic regulation, to provide useful information for understanding the cellular molecule interaction and disease etiology.

## 2. The Genome Architecture of LncRNAs

The definition of lncRNA is the transcript that is longer than 200 nucleotides and without the ability of encoding proteins. However, it would be too narrow and absolute if only the common feature of length is used to classify lncRNAs from each other. According to the genome location, sequence, morphology, structure, and function features, lncRNAs can be categorized into different groups. LncRNAs can be divided into intergenic lncRNAs (lincRNAs) and intronic lncRNAs, from the level of genome location, which occupy 98–99% of the human genome [14]. Another classification type is considered regarding the product orientation of the DNA strand and divides lncRNAs into sense ones and antisense ones. The focused research types are concentrated into lincRNAs and antisense lncRNAs, especially for the crosslink of antisense lncRNAs and lincRNAs [15,16,17]. Other classification standards depend on whether it is associated with a known DNA element, by which it can be divided into enhancer associated lncRNAs, promoter associated lncRNAs, upstream antisense RNA, and telomeric repeat RNA. Depending on whether the protein coding gene is related, it can be divided into natural antisense RNA and cyclic annular intronic gene, etcetera [18]. Along with the development of lncRNA annotation studies, the new classification is apt to introduce the function and structure information of lncRNA to enhance the category stability. The concept of an RNA family is used to stand for the functional and structural similarity of lncRNAs [19].

**Figure 1:**
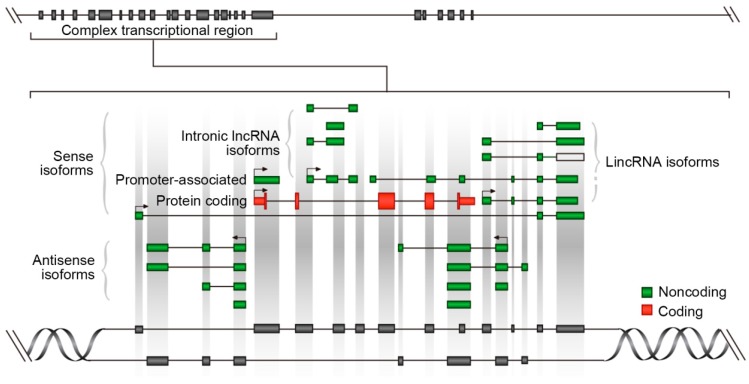
LncRNA architecture in complex transcriptional region.

Each type of lncRNAs has its own complex and specific location. The location will sometimes determine the function context of the lncRNA [20]. Figure 1 shows a slightly complex genome partition, with sense and antisense non-coding sequences nested with coding sequences. LncRNAs that are adjacent to coding genes or clustering with coding genes are comprehensively studied, and will provide some functional evidence for lncRNAs to annotate the mechanism of transcriptional and epigenetic regulation. Many studies focused on the lncRNAs localization and function association. The human cancer related transcription factor *MYC* coding gene is located in the region of 8q24, which is surrounded by a number of non-coding regulatory elements [21]. The 515 kb upstream of the *MYC* gene has a *CCAT1* non-coding region on it, which mainly includes two types of lncRNA: CCAT1-S and CCAT1-L. Recent research has shown that CCAT1-S and CCAT1-L are highly specific markers in some tumor diseases, and knock down of the two loci can reduce the expression of the *MYC* locus. Therefore, it suggests that CCAT1-S and CCAT1-L have potential *Cis*-regulation activity on the *MYC* locus [22].

## 3. The Epigenetic Regulatory Activity of lncRNAs

LncRNA shows strong epigenetic function roles by the direct regulation or indirect interaction with other molecules [23,24]. LncRNAs exhibit epigenetic characteristics that are similar to coding genes, such as maternal effect, DNA methylation and histone modification activity, as well as post-transcriptional regulation. H19 is a length of 2.3 kb of lncRNA, and only found in maternal expression [25]. It is highly expressed during the development of vertebrate embryos, and is rapidly down-regulated in most tissues after birth [26]. During the pathological mechanism study of gastric cancer, it was found that lncRNA GClnc1 could be used as a molecular scaffold to recruit WDR5 and KAT2A complexes for specific modifications of histones [27,28]. Another example is that HOTAIR (HOX Transcript Antisense RNA) forms at least two histone modifying complexes that are involved in histone modification reactions [29,30]. Additionally, lncRNAs have been proven to participate in the methylation process of CpG islands. Since DNA methylation is suggested to be close to gene proliferation, lncRNA is thought to play a significant role in epigenetic regulation in human cancer and other diseases [31]. A lot of evidence also shows that lncRNA plays important roles in X chromosome inactivation, gene imprinting, and gene silencing [32,33]. LncRNA Airn, with a length of 108 kb, is transcribed by paternal alleles, which causes paternal specificity silencing by *cis*-regulation. Airn is expressed at a specific imprinting site, recruiting G9a to H3K9 methylation residues, and silencing *IGF2R*, *SLC22A3* and *SLC22A2* genes in the genome by a *cis*-regulation manner over paternal origin of 300 kb [34]. Sometimes, lncRNA would even appear in the form of targeting vectors or the host of epigenetic regulated factors [35,36]. The typical cases are competing endogenous RNAs (ceRNAs) and the recently found tRNA-derived small RNAs (tsRNAs). The former is a large group of lncRNAs that can competitively bind microRNAs with mRNA, and the latter is proven to be transported into a fertilized egg by sperms and present an intergenerational epigenetic effect [36].

The molecular biological research and CLIP-sequence data analysis shows that lncRNAs can interact with a variety of protein molecules, and implies a potential mechanism of disease etiology. The clip-db database stores 111 RNA binding proteins (RBPs) of 395 available clip datasets from human, mouse, worm, and yeast [37]. These data are only the tip of the iceberg, and will be multiplied soon. The interaction disorder of RNA-protein will get further attention, too. LncRNA P21 (linc-p21) shows an important role in the P53 pathway and activated by P53 for cell apoptosis. Transcriptional repression is also implemented by binding to ribonucleoprotein-K (hn-RNP-K) [38]. MALAT1 is highly expressed in breast, colon, and prostate, and is shown to interact with the splicing regulation factor (SR protein). The exceptional situation of RNA-protein interaction will lead to serious illness [39,40].

LncRNAs can also interact with RNA molecules, such as mRNA, miRNA, ceRNA, which is another type of post-transcription regulation. Multiple classes of lncRNA can interact with each other to alter the gene expression abundance or transcription isoforms, as ceRNA is described previously. The negative correlation between lncRNA specific transcript GAS5 and miR-21 are that they suppress each other’s expression [41]. Zhang Z. and colleagues, in the 83 human disease-related lncRNAs real time PCR studies, find that miR-21 is capable of suppressing the expression of GAS5 by targeting on the binding site in exon 4. Zhang et al. also find that GAS5 can repress miR-21 expression. It is proven that miR-21 expression will increase, when the expression of GAS5 is suppressed. As an important type of ceRNA, circular RNA (circRNA), has received extensive attention. It often appears as a molecular sponge to perform functions by post-transcriptional regulation. A large number of tools to detect the cirRNAs and their probes have been developed, which provide useful information for illuminating the biological mechanisms [42]. Another example shows that the different isoforms of lncRNA-MIAT expressed in meiotic cells and mitotic anaphase retinal cells can regulate microvascular dysfunction [43].

## 4. The Structural Basement of LncRNA in Epigenetic Regulation

Accumulating studies indicate that lncRNA structure is one of the most critical factors to perform function. LncRNA secondary and higher structure is of great significance for exploring the RNA molecular mechanism in the biological processes, such as the interaction between RNA and bio-macromolecules, the characteristics of RNA family classification, and more [44,45,46,47]. Previous studies found multiple sequence alignment, which implied that lincRNA sequences are relatively conserved in certain RNA families, while they are not well-conserved for the total lncRNAs. However, in the lncRNA structure studies, the trend is to be evolutionarily conserved across different species resulting in a similar expression and function [48].

LncRNAs secondary structure is folded naturally through the approximate minimum free energy mode and is affected by cell situation and inner environment. The secondary structure, resulting from RNA interaction, includes stem, hairpin, bulge loop, inner loop, multi-branch loop, pseudoknot, etcetera [49]. These secondary structures are related to each other and form tertiary structure by further complementation of Watson—Crick base pairing, and leads to the fact that the RNA structure is coaxial through the double helix, in a parallel or vertical manner [50]. Some lncRNA structure forms modular features that are accompanied by a periodic motif, such as a sarcin-ricin loop, K-turn, and C-loop [51]. The structure of lncRNA has plasticity and enables itself to participate in many functions, such as organization, catalysis, and regulation. Long strand RNA transcripts are more flexible and plastic, so they may have more complex structures effective for molecular interaction and dynamic regulation, and obtain the functions that are acting as the switch of the reaction, the basement of the protein, and forming a structure motif of specific regulation [52].

To understand the relationship between the structure and function of lncRNA, it is necessary to recognize the structure of RNA accurately. The methods of exploring RNA structure are mainly through bioinformatics algorithms, biochemical methods, and enzyme-probe based RNA structurome methods [53,54,55]. The computational prediction of the secondary structure is based on two viewpoints: the minimum free energy model and the multiple sequence alignment [56]. Mainstream prediction software for the secondary structure prediction, include Pfold, RNAfold, RNAstructure, and more [19,57,58,59]. The toolkits for identifying the tertiary structure contain FaRNA, NAST, 3DRNA, and so forth [60,61,62]. LncRNA structure prediction, with the development of RNA-sequence and high-throughput sequencing, comes into the era of structurome, and the efficiency and accuracy will be improved [63,64]. Recently, a great deal of significant progress has been made in the study of RNA modification, especially for the *N*6-methyladenosine (m6A). Series of important computational methods, such as Ensemble Support Vector Machines, improve the detecting level, and will further deepen the understanding of RNA structure [65].

Some progress has been made in the study of the correlations between the structure and function of lncRNAs, and some useful evidence to explain the complex regulation mechanism in the cell cycle and disease genesis has been found. The 2.2 kb length lncRNA HOTAIR has four sub domains, two of which have been confirmed to be highly conserved protein binding regions [66]. These structured regions play important roles in the function achievement of HOTAIR, and the structure motif features obtain the computational research certification. LncRNA MEG3 is proven to have the ability to suppress cancer, and the functional maintenance is dependent on the structural characteristics [67]. The recent study shows that the main component of human hormone receptor co-activator is lncRNAs SPA1. Through chemistry and enzyme analysis, four distinguishing structure domains are found on SPA1 and keep the functional activity [68]. The development of the experimental and computational technology of RNA structure identification will enable specific descriptions of the RNA structure to be possible, and more accurate lncRNAs correlations between structures and functions will be obtained [69,70].

## 5. The Models of LncRNA Structure Mediated Epigenetic Regulation

The features of lncRNAs, such as diversity, structuring, conservation, and plasticity, determine their complex functional roles in the biological process [71]. The variability, flexibility, and variety of structural features of lncRNAs in the genomic dynamics lead them to be significant components in epigenetic regulation [72]. A series of studies present that lncRNA structure induced epigenetic regulatory mechanisms are accomplished mainly by three different processes. First, it could use the allostery effect to match different regulation proteins. Second, it could act as a molecular scaffold to recruit chromatin modifying proteins. Third, it could use repeat elements to affect the expression of their surrounding genes [73,74].

**Figure 2:**
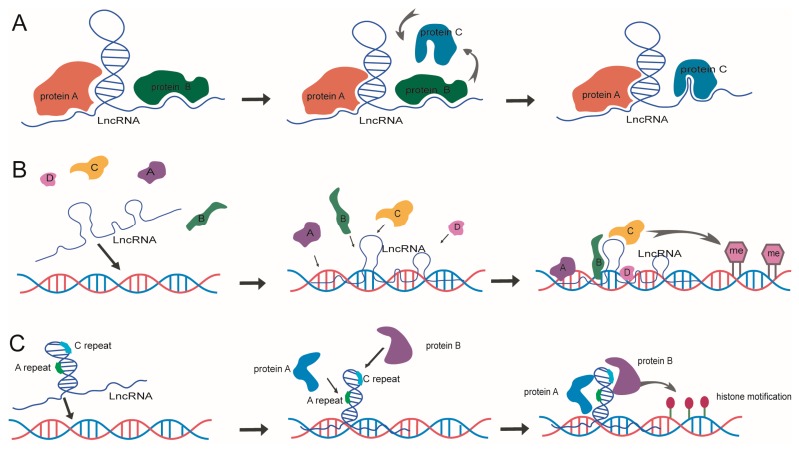
The models of LncRNA structure mediated epigenetic regulation. (**A**) LncRNA allostery effect for interacting with different ligand proteins. (**B**) LncRNA acting as molecular scaffold to recruit and combine with multiple regulatory proteins. (**C**) LncRNA mediating histone modification by the functional region repeat elements.

Allostery, which is determined by relatively active chemical attributes, is one of the most important characters for lncRNA to perform the epigenetic regulatory function. It will alter the structure of lncRNA to bring about or take off the structural binding regions of proteins in a certain space and time situation (Figure 2A). LncRNAs can fold regularly into some different structures, which gives the opportunity to lncRNA to carry out the function of molecular switching and change the activation of genome regions [75]. A certain cellular environment can have HOXC clusters, originated as HOTAIR, which are able to change its conformation and combine with the PRC2 factor to target the *HOXD* clusters by a *trans*-regulating manner, then inhibit the histone modifications [76,77,78]. The longer sequences show lncRNAs tend to keep a large number of protein binding sites, when compared with other RNAs, which provide a much wider space for the allostery effect to affect a series of protein interactions and molecular regulations [79]. The allostery effect also can be induced by the polymorphisms or mutations of lncRNAs, which frequently presents in the physiological or pathological processes [80,81].

LncRNA, acting as molecular scaffolds, is introduced by multiple structure motifs [74]. These motifs have the ability to recruit multiple proteins to combine with the lncRNA by forming stable ribonucleoproteins (Figure 2B) [82]. Two-thirds of the components for the ribosome of eukaryotic cells are noncoding RNAs, and these RNAs consist of two subunits. Approximately 86 proteins combine with a 1.9 kb and a 4.7 kb region of two RNA strands, which makes ribosomes exhibit a highly structured and rigid scaffold. LncRNA CDKN2B-HS1 can recruit the Suz12 subunit of PRC complexes and CBX7 subunit of PRC1 complexes to combine with the H3K27 modification locus and mediate the suppression of the expression of cancer associated *CDKN2A*/*CDKN2B* genes [83]. Some evidence shows that the chromatin modifying proteins have the ability to bind with different lncRNAs, and some lncRNAs can form structures in different regions of itself to bind multiple proteins, so as to perform their function for histone modifications [84]. There are two functional structure motifs at the 5′ and 3′ end of HOTAIR. The 5′ end motif has the ability to connect to the methyltransferase activity fragment EZH2 of the PRC protein. The 3′ end has the ability to recruit lysine demethylases. The combination of these two motifs will lead to catalyzing demethylation of H3K4 and achieve the objective of gene expression regulation [29]. More and more new epigenetic regulatory mechanisms that are similar to the RNA scaffold have been found. Some lncRNAs can also perform their functions by acting as a *cis*-functional element, and a *trans*-regulatory factor, simultaneously. The lncRNA can bind with the promoter of dihydrofolate reductase *DHFR* and form the DNA–RNA three-helix structure to suppress the gene expression. Correspondingly, it could directly bind to the transcription factor TF2B to prevent the formation of transcriptional initiation complexes and interfere with the *DHFR* transcription [85].

Another effect of the LncRNA structure affecting epigenetic regulation is the functional region repeat elements, which will induce proteins and RNAs to perform the inactivity of gene and chromatin (Figure 2C). Xist contains several modular structure regions, including A-repeat and C-repeat, each of which has its own function in X chromosome activity regulation. The A-repeat region, which is located at the 5′ end, contains nine repeating elements and forms two stem loop structures to recruit PRC1 combination. One of the stem loops is the AUCG hairpin loop, which is extremely important for Xist to perform its function for X chromosome silencing [79]. The C-repeat region combines with hnRNPU and YY1, to participate in the interaction of Xist and the X chromosome. Another typical case is lncRNA Rsx. Although Rsx has evolved independently and has no homology with Xist, it has the similar function to Xist by sharing the functional enrichment repeat sequences, repeat elements, and conserved stem loop motif at the 5′ end [86,87,88].

## 6. Summary and Expectation

LncRNAs have been extensively studied, and many new techniques have been applied to lncRNA sequencing and structure analysis, including some genome-wide strategies. Numerous evidence indicates that the structural features of lncRNAs are crucial to understanding their functions and roles in physiology and pathology. This article mainly discusses the lncRNA structural and functional activity in the epigenetic regulation process. It shows that diverse and complex progress is contained in the level of different epigenetic regulatory styles, such as methylation, histone modification, and gene imprinting. It also presents the overall perspective of the structural and functional features of lncRNAs in the epigenetic regulatory process, especially the key mechanism that is induced by allostery, scaffold, and repeat elements, in the current research situation.

Although, the time and number of the lncRNAs structure and function correlation research in epigenetic regulation is limited, and most of the accurate lncRNA structure and function should be further annotated, the study of the lncRNA epigenetic effect has huge potential for a breakthrough in cellular and disease research. More effort is needed, at least in the field of regulatory protein binding site identification, the functional sequence motif screening, the lncRNA leading multiple factors synergistic regulation, and the lncRNA allostery mechanism in different advances of molecular interaction, by which a full prospect will be presented in the future.

## Figures and Tables

**Table 1:** The representative epigenetic regulation functions of long non-coding RNAs (lncRNAs).

LncRNA	Function	Reference (PMID)
Airn	Paternal specificity silencing	10988110
CCAT1-L	Influence the expression of the *MYC* locus	27147598
CCAT1-S	Influence the expression of the *MYC* locus	26254776
CDKN2B-HS1	Suppression of the expression of cancer associated *CDKN2A/CDKN2B* genes	20541999
GAS5	Suppress the expression of miR-21	20041488
GClnc1	Recruit WDR5 and KAT2A for specific histone modification	26289363
H19	Maternal expression, rapidly down-regulated in most tissues after birth.	28930564
		20486113
HOTAIR	Form multiple histone modifying complex involved in histone modification reactions	20616235
		25866246
Linc-P21	Play important role in the P53 pathway and activated by P53 for cell apoptosis	20673990
MALAT1	Highly expressed in breast, colon, and prostate	24297251
	Associated with many diseases	22425269
MIAT	Regulate microvascular dysfunction	25587098
MEG3	Suppress cancer	20032057
Rsx	Regulate the X chromosome silencing	12649488
SPA1	Hormone receptor co-activator	22362738
XIST	Regulate the X chromosome silencing	21947263
		11780141
		20833368
